# Inference of Gene Regulation via miRNAs During ES Cell Differentiation Using MiRaGE Method

**DOI:** 10.3390/ijms12129265

**Published:** 2011-12-12

**Authors:** Masato Yoshizawa, Y-h. Taguchi, Jun Yasuda

**Affiliations:** 1 Department of Physics, Faculty of Science and Engineering, Chuo University, 1-13-27 Kasuga, Bunkyo-ku, Tokyo 112-8551, Japan; E-Mail: masa.yoshizawa44@gmail.com; 2 Tohoku University School of Medicine, 1-1, Seiryo-machi, Aoba-ku, Sendai 980-8574, Japan; E-Mail: yasuda-jun@umin.ac.jp; 3 Department of Cell Biology, the JFCR-Cancer Institute, 3-8-31, Ariake, Koto-ku, Tokyo 135-8550, Japan

**Keywords:** miRNA, embryonic stem cell, MiRaGE metheod

## Abstract

MicroRNA (miRNA) is a critical regulator of cell growth, differentiation, and development. To identify important miRNAs in a biological process, many bioinformatical tools have been developed. We have developed MiRaGE (MiRNA Ranking by Gene Expression) method to infer the regulation of gene expression by miRNAs from changes of gene expression profiles. The method does not require precedent array normalization. We applied the method to elucidate possibly important miRNAs during embryonic stem (ES) cell differentiation to neuronal cells and we infer that certain miRNAs, including miR-200 family, miR-429, miR-302 family, and miR-17-92 cluster members may be important to the maintenance of undifferentiated status in ES cells.

## 1. Introduction

miRNA is a short (19~22 base) non-coding RNA, which is known to regulate gene expression during cancer formation, lineage differentiation and some other biological processes [1]. There are approximately 1000 distinctive miRNAs in the human genome and more than 60% of human genes are regulated by miRNA [2].

The miRNA can suppress multiple messenger RNAs (mRNAs) which have target sites in their 3′ untranslated regions (3′ UTRs). By base pairing with its “seed sequence” at the 2nd–8th base of 5′ end, miRNA interacts with 3′ UTR of target mRNAs in a miRNA-protein complex called miRISC and suppress the gene expression by suppression of translation and/or degradation of target mRNA. Several reports indicate that the majority of gene suppression induced by miRNA is the degradation of target mRNA [1].

Based on the nature of target recognition mechanism by miRNA (base pairing at seed sequence) and its primary functional outcome (degradation of target messengers), it may be possible to infer which miRNA may play major roles during a biological process from the difference of mRNA expression profiles. Based on this notion, we developed a method for inference of miRNA regulation from target gene expression profiles and computationally expected miRNA target databases [3,4]. We named the new method as MiRaGE (MiRNA Ranking by Gene Expression). Similar algorithms have been developed and available in public, such as T-REX [5], SylArray [6], and MirACT [7]. MiRaGE is focused on the direct targets of miRNAs for the analysis and normalization procedures between arrays are included in the algorithm and therefore one can omit array normalization.

In this paper, we have applied MiRaGE method to gene expression profile during differentiation from mouse embryonic stem (ES) cells. ES cells have pluripotency and their molecular mechanisms of cell differentiation are extensively studied. It is reported that miRNAs play critical roles in the maintenance of stemness in ES cells as well as in the differentiation to the multiple cell lineages [8]. We applied the MiRaGE method to transcriptome data sets during differentiation of mouse ES cells available in public and found several miRNAs such as miR-200 family, miR-429, miR-302 family, and miR-17-92 cluster members, which seem to be important to the maintenance of undifferentiated status in ES cells.

## 2. Results and Discussion

### 2.1. Development of MiRaGE Method

**Figure 1:**
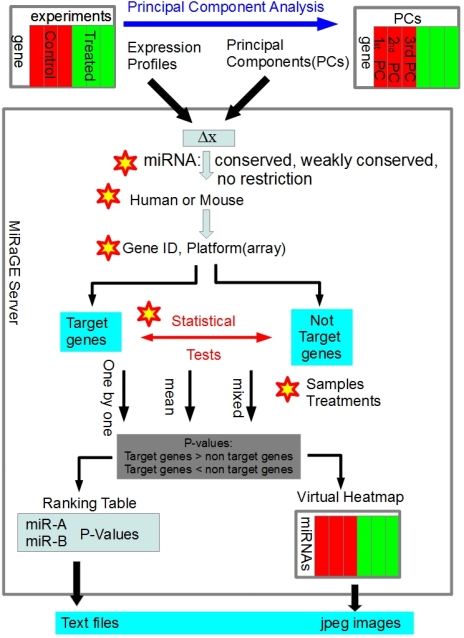
Schematic diagram of MiRaGE method. Gene expression profiles of samples should consist of control and treated (presented in red and green, respectively), each of which can include more than one biological/technical replicates. The difference/logarithmic ratio **Δ*x*** between control and treated is computed from gene expression profiles or principal component scores (see main text). The server provided several choices of analytical parameters (indicated with asterisks). Output files are a miRNA ranking with *P*-value in text format and the virtual heat map of miRNAs as a jpeg file. In some cases, it is useful to apply principal component analysis to gene expression profiles prior to the analysis. In this case, component scores for each sample can be substituted for **Δ*x*(*g*)** (see Materials and Methods).

The outline of the MiRaGE server [9] is indicated in Figure 1 and the methodological detail has been described in [3,4]. When miRNA *m* is considered, genes are classified into three categories: (1) target genes of *m*; (2) non-target genes of *m* but targeted by any other miRNAs; (3) genes not targeted by any miRNAs. Hereafter, we denote the set of genes which belong to category (1) as *G_m_* and the set of genes *G*_0_*_m_* includes genes in category (2). Then the statistical significance of difference in gene expression between *G_m_* and *G*_0_*_m_* for each miRNA is calculated with various methods.

We have already established a web server [9] to perform MiRaGE method. MiRaGE server computes these *P*-values employing either Kolmogorov-Smirnov test, Wilcoxon rank-sum test, or Student *t*-test. Three sets of miRNAs can be analyzed: conserved, weakly conserved, or no restriction. Then *P*-value is compared to check if miRNA *m* significantly regulates its target genes between two different experimental conditions. After applying FDR correction (BH method [10]) to 162 *P*-values, we have selected *m*s whose FDR corrected *P*-values are less than 0.05 as miRNA which regulates target genes significantly.

### 2.2. miRNAs Underrepresented in ES Cells after the Differentiation to Neuronal Cells

Aiba *et al.* [11] measured gene expression profiles of mouse ES cells during differentiation to several lineages. Among them, we have analyzed gene expression profile during the differentiation to neuronal cells with N2-supplement B medium. They have measured six time points: each day from 0 to 5 days during differentiation induction. Importantly, they added Universal Mouse Reference RNA supplemented with mRNAs of ES cells as internal references (UMRR + ES), labeled with Cy5. All sample RNAs were labeled with Cy3 and mixed with Cy5-labeled UMRR + ES and subjected to microarray analyses. Addition of UMRR + ES was intended to precise quantification of each gene expression in regards to the externally added reference RNA [11]. Since there are two biological replicates for each time point, we can have in total 2^6^ = 64 distinct combinations of time points. After applying principal component analysis (PCA) to total 64 combinations of normalized profiles, we have uploaded all of the first PCs to MiRaGE server. Since the first PCs turn out to be monotonic decease/increase of gene expression as time goes (data not shown), we may identify miRNAs which effect on the gene expression profiles in a monotonic manner.

Table 1 indicates the miRNAs underrepresented in ES cells after induction of differentiation to the neuronal cells by MiRaGE method. In other words, these miRNAs may be down regulated in ES cells after differentiation and important for the stemness of ES cells. There are many miRNAs known to be biologically critical in ES cell biology appeared in Table 1. For instance, miR-302a/b/d, miR-290 cluster (291a-3p, 294 and 295), miR-200, and miR-429 are reported to be upregulated in undifferentiated ES cells [8,12]. Recently, induced pluripotent stem cell can be generated with several miRNAs including miR-302 and miR-200 [13,14]. It is also remarkable that miR-106a/b are listed in miRNAs dominated in ES cells [15]. Most of the above mentioned miRNAs showed statistical significance in all three analyses used in MiRaGE method. The MiRaGE could infer many miRNAs which are believed to be critical in stemness in ES cells. These results revealed the power of MiRaGE method for inference of important miRNAs in biological processes.

As an analytical method, the MiRaGE can accept the principal component scores (PCs) of the expression profiles (see Figure 1 and section 3). It gives us an interesting possibility to MiRaGE for inference of miRNAs specific in a PC. In the other words, if a PC is related to a particular subpopulation in the sample population, we can infer specific miRNAs important for the subpopulation. In addition, changing statistical methods does not drastically affect the list of selected miRNAs (Table 1). This suggests the robustness of the MiRaGE method.

To test whether these highly ranked miRNAs as underrepresented in ES cell differentiation, we analyzed another data in the same dataset: the differentiation to trophoblast from ES cells (Table 2).

Table 2 indicates the miRNAs that mostly appeared in the top 50 underrepresented in ES cells between neural and trophoblast differentiation [10]. Among them, 50% (25) of miRNAs are commonly underrepresented and all the above mentioned miRNAs appeared in the list. Among the 25, we found miRNAs that belong to the miR-17-92 cluster (miR-20, 93, and 17). The miR-17-92 cluster is known to be a critical component in the MYC pathway [16] and contributes to tumorigenesis in several malignancies [17]. It is a very interesting possibility that the cluster miRNAs may contribute the stemness of non-tumor pluripotent stem cells.

### 2.3. miRNAs May Be Overrepresented in ES Cells after the Differentiation to Neuronal Cells

There are many miRNAs known to be critical in neurogenesis. We had expected that MiRaGE could find some of those characterized miRNAs in the dataset of Aiba *et al.* [11]. However, we could not statistically infer significant miRNA as overrepresented after the neuronal differentiation (*i.e.*, potentially critical in neural differentiation) when we tried the standard MiRaGE method. To overcome this difficulty, we changed the algorithm of data processing as described in the method section and found three miRNAs showed relatively small *P*-values in *t*-test (Table 3).

Among them, miR-184 is known to be overexpressed in the central nervous system [18], indicating the biological relevance of the MiRaGE. However, we could not see the well-documented miRNAs for neurogenesis such as miR-9 or miR-124 [19]. To address this issue, we have analyzed the other dataset by the same group [20] with the modified MiRaGE method. The comparison between ES and adult brain tissue indicated that miR-124 showed modest significance as overrepresented in brain (*P* = 0.044), suggesting that the miRNA may be more important in the mature brain tissue than neuronal-differentiating ES cells.

Generally, most of the analyzed miRNAs showed strong statistical significance as underrepresented in differentiating ES cells, whereas not many miRNAs were statistically significant as overrepresented in our analysis (Tables 1 and 3). We have analyzed the difference of gene expression of the miRNA targets with the same data set and more than half the probes of whole miRNA targets used in MiRaGE analysis were downregulated in ES cells (53.5%, *P* < 0.0001). We analyzed the other data set using a different group: ES cell differentiation to the Embryonic body [21]. The results showed that much less miRNA targets were downregulated in ES cells compared with differentiated cells to embryoid bodies (23.6%, *P* = 0.007) and we did not observe the uneven distribution of *P*-values in the MiRaGE analysis with the dataset (data not shown). Hence the uneven distribution of *P*-value shown in Tables 1 and 3 may be caused by the experimental procedure [11].

Based on the statistical algorithm, the number of suppressed target mRNA species, rather than the absolute suppression of target gene expression, achieves lower (*i.e.*, more significant) *P*-values using the miRNAs in the original MiRaGE method. This characteristic of the original MiRaGE is apparent in the difference of *P*-values shown in Tables 1 and 3. The biological logic of this algorithm is based on the notion that the miRNA with more target species should be biologically more important. As mentioned above, we had to modify the analytical method of MiRaGE to see the known critical miRNA in neural differentiation as high-ranked ones. This experience suggested that some miRNAs may function by suppression of fewer targets with the larger absolute differences of mRNA expression. That type of miRNAs may be more difficult to significantly infer using the original MiRaGE method as the absolute differences of mRNA expression is not used in the analysis. Further experimental and analytical studies will be needed to define which calculation method of MiRaGE may be more suitable to individual types of datasets and/or miRNAs.

The present study showed the potential of the MiRaGE method in the prediction of miRNAs critical for cellular differentiation. MiRaGE at present, however, may be more suitable for the inference of differently functioning miRNAs between two quite similar but distinct cell populations such as histopathological subtypes in various human cancers. For example, ovarian cancers consist of several different morphological subtypes (serous, mucinous, endometrioid, clear cell and undifferentiated) and some of them show poorer prognosis. MiRaGE may be useful to find out the specific miRNAs for poor prognostic subtypes and give an insight for the molecular therapeutic targets of the cancers.

How then, can we improve the MiRaGE method to analyze the difference of miRNAs functions between the two datasets with quite different expression profiles? One possibility is by analyzing the datasets of gene expression profiles with miRNA overexpression or suppression. This type of experiment reveals important parameters for MiRaGE such as valid targets of miRNAs in a cell and the extent of maximum gene suppression by the miRNAs for each target. We can adjust the strength of the miRNAs in suppression of each target gene expression more precisely. We may give further additional parameters such as the state of chromatin modifications in the promoter regions of target genes in the two different states of the cells; we may delete target genes whose promoter is inactivated in a particular cell. Curating of potential targets by experimental data and promoter activities may also improve the precision of the MiRaGE method.

## 3. Materials and Methods

### 3.1. Detailed Method of MiRaGE

Suppose that ***x***(***g,e***) is the expression of gene ***g*** at the experiment ***e***. The genes in the set ***G****_m_* are denoted as ***g****_m_* and those in ***G*_0_***_m_* as ***g*_0_***_m_* (see main text for ***G****_m_* and ***G*_0_***_m_*). The control experiment is denoted as ***e****_cntl_* and the treated experiments is as ***e****_treated_*. First, we compute the logarithmic ratio Δ***x*** of gene ***g*** as

(1) $$\Delta x(g)=\text{log}\left[\frac{x(g,e_{treated})}{x(g,e_{cntl})}\right]$$

or the difference

(2) $$\Delta x(g)=x(g,e_{treated})-x(g,e_{cntl})$$

For principal component analysis, we have taken the coordinate of each gene along the 1st principal component as Δ***x***(***g***).

*P*-value which reject the null hypothesis to the alternative hypothesis, that is, target genes of miRNA *m* is overrepresented than non-target genes, is indicated as

(3) $$P(\{\Delta x(g_{m})\}>\{\Delta x(g_{0m})\})$$

Similarly, *P*-value which rejects the null hypothesis to the alternative hypothesis, that is, target genes of miRNA *m* is underrepresented than non-target genes, is indicated as

(4) $$P(\{\Delta x(g_{m})\}<\{\Delta x(g_{0m})\})$$

If we have *n* and *n*’ replicates for control and treated experiments, we compute *P*-values for all of all *nn*’ pairs of experiments.

### 3.2. Inference by MiRaGE Server for the Differentiation from ES Cell

Application of MiRaGE method (Figure 1) has been done by MiRaGE server [9]. It provides the following analytical parameters as pull-down menu choices to compare target/non-target genes: the set of miRNAs for inference (conserved, weakly conserved, or no restriction), species (human or mouse), types of gene identification (several gene IDs or microarray probe IDs), statistical tests (Kolmogorov-Smirnov test, Student *t*-test, or Wilcoxon test), and calculation method (one by one, mixed, or mean). The calculation methods for *P*-values to reject the null hypothesis (the target and non-target genes are not differently expressed) are computed in the three ways: one by one (MiRaGE for all possible pairs between control and treated experiments independently and medians of the *P*-values are used as representatives), mixed (all of control or treated samples are handled as one set for MiRaGE), or mean (mean values of gene expression among control or treated samples are used for MiRaGE calculation). The parameters set as following; “Select type of identifier” is set to be RefSeq. “Select Statistical test” is tested all three (Kolmogorov-Smirnov test, *t*-test, and Wilcoxon. test). “Select how to treat samples” is set to be “one by one” such that we can get all of combinations between controls and treated. Other options including “conservation of miRNA” remain as default. Thus, we consider only 162 conserved miRNAs for the analyses. The coordinate itself of each gene along the 1st PC is taken to be Δ***x***(***g***) as mentioned in Sec. 3.1.

We have improved the MiRaGE method from our previous studies [2,3]. First, the target gene tables are generated by us. We have downloaded sequences of 3′ UTR of all RefSeq genes using genome browser table [22] and miRNA sequences from miRbase [23]. Then, we have counted seed match between 3′ UTR of RefSeq genes and 2nd to 8th bases from 5′ end of miRNAs. Based upon this count, genes having at least one seed match are selected as target genes of the miRNA. Secondly, we have added the option for selecting statistical methods other than *t*-test.

To obtain *P*-values to get ranking in Table 3, we calculated all of the ratio of each Cy3 signal with corresponding Cy5 signal and the relative expression profiles of each gene for ES cells and ES cells induced neuronal differentiation for five days to MiRaGE server. Then we have picked up miRNAs which are ranked as top 30 among 162 microRNAs analyzed. We calculated the difference (1) instead of the logarithmic ratio (2) between day 0 and day 5 without applying PCA. Other parameters of the analyses were same as above.

### 3.3. Principal Component Analysis (PCA)

Principal component analysis is a method to reduce the dimensionality of multiple, possibly correlating variables into new sets of uncorrelated variables as principal components (PCs) and it has been applied to the gene expression profiling analysis (see an example in [24]). The information of the dataset can be summarized into the series of PCs. PCs are ordered variables as first PC has the largest information of the original dataset. To generate the PCs, the orthogonal transformation using the correlation among the variables in data matrix is serially applied to the dataset and PC is generated at each step of transformation. We calculated the PCs of the dataset of gene expression profiles by the use of R packages (prcomp or princomp function in the base package).

By applying PCA to gene expression from ES cell to neuronal cell/trophoblast, we have found that monotonic decrease/increase is the most significant component (*i.e.*, the first principal component) to express gene expression diversity. Thus, we employ it as the representative of gene expression profile during the differentiation from ES cell to neuronal cell/trophoblast.

## 4. Conclusions

In this paper, we have applied the MiRaGE method to differentiation of ES cell to neuronal cells to infer biologically critical miRNAs in ES cell stemness or neuronal differentiation. With some modifications to the methods, we can successfully list biologically critical miRNAs during differentiation from ES cell to neurogenesis and the present study revealed potential and suitable applications of the MiRaGE method as an analytical tool for the study of miRNA functions.

## Figures and Tables

**Table 1:** Top ranked miRNAs underrepresented in ES cells after differentiation to neuronal cells.

miRNA underrepresented after neural differentiation	*P*-values		

	KS	*t*	Wilcoxon
mmu-miR-200b	4.14 × 10^−16^	9.17 × 10^−17^	5.39 × 10^−17^
mmu-miR-200c	4.14 × 10^−16^	9.17 × 10^−17^	5.39 × 10^−17^
mmu-miR-429	4.14 × 10^−16^	9.17 × 10^−17^	5.39 × 10^−17^
mmu-miR-291a-3p	2.43 × 10^−13^	5.88 × 10^−15^	1.36 × 10^−16^
mmu-miR-294	2.43 × 10^−13^	5.88 × 10^−15^	1.36 × 10^−16^
mmu-miR-295	2.43 × 10^−13^	5.88 × 10^−15^	1.36 × 10^−16^
mmu-miR-302a	2.43 × 10^−13^	5.88 × 10^−15^	1.36 × 10^−16^
mmu-miR-302b	2.43 × 10^−13^	5.88 × 10^−15^	1.36 × 10^−16^
mmu-miR-302d	2.43 × 10^−13^	5.88 × 10^−15^	1.36 × 10^−16^
mmu-miR-141	4.66 × 10^−13^	3.08 × 10^−11^	1.68 × 10^−11^
mmu-miR-200a	4.66 × 10^−13^	3.08 × 10^−11^	1.68 × 10^−11^
mmu-miR-23b	2.18 × 10^−12^	1.30 × 10^−14^	7.32 × 10^−16^
mmu-miR-23a	2.18 × 10^−12^	1.30 × 10^−14^	7.32 × 10^−16^
mmu-miR-30a	1.02 × 10^−11^	3.09 × 10^−10^	3.96 × 10^−10^
mmu-miR-30b	1.02 × 10^−11^	3.09 × 10^−10^	3.96 × 10^−10^
mmu-miR-30e	1.02 × 10^−11^	3.09 × 10^−10^	3.96 × 10^−10^
mmu-miR-30c	1.02 × 10^−11^	3.09 × 10^−10^	3.96 × 10^−10^
mmu-miR-30d	1.02 × 10^−11^	3.09 × 10^−10^	3.96 × 10^−10^
mmu-miR-384-5p	1.02 × 10^−11^	3.09 × 10^−10^	3.96 × 10^−10^
mmu-miR-129-5p	4.31 × 10^−11^	8.12 × 10^−11^	7.63 × 10^−11^
mmu-miR-218	1.06 × 10^−10^	3.11 × 10^−11^	1.33 × 10^−11^
mmu-miR-144	1.75 × 10^−10^	8.24 × 10^−11^	1.34 × 10^−10^
mmu-miR-130a	3.36 × 10^−10^	1.20 × 10^−8^	3.35 × 10^−8^
mmu-miR-301a	3.36 × 10^−10^	1.20 × 10^−8^	3.35 × 10^−8^
mmu-miR-130b	3.36 × 10^−10^	1.20 × 10^−8^	3.35 × 10^−8^
mmu-miR-301b	3.36 × 10^−10^	1.20 × 10^−8^	3.35 × 10^−8^
mmu-miR-721	3.36 × 10^−10^	1.20 × 10^−8^	3.35 × 10^−8^
mmu-miR-135a	4.83 × 10^−10^	1.91 × 10^−10^	6.04 × 10^−10^
mmu-miR-135b	4.83 × 10^−10^	1.91 × 10^−10^	6.04 × 10^−10^
mmu-miR-106a	5.35 × 10^−10^	4.36 × 10^−9^	1.98 × 10^−8^

KS: Kolmogorov-Smirnov; *t*: Student *t*; Wilcoxon: Wilcoxon rank-sum test.

**Table 2:** Commonly underrepresented miRNAs in ES cells during differentiation to neuronal cells or trophoblast.

miRNA underrepresented after differentiation	*P*-values *		

	KS	*t*	Wilcoxon
mmu-miR-106a	1.07 × 10^−15^	6.83 × 10^−13^	9.39 × 10^−15^
mmu-miR-106b	1.07 × 10^−15^	6.83 × 10^−13^	9.39 × 10^−15^
mmu-miR-20a	1.07 × 10^−15^	6.83 × 10^−13^	9.39 × 10^−15^
mmu-miR-93	1.07 × 10^−15^	6.83 × 10^−13^	9.39 × 10^−15^
mmu-miR-17	1.07 × 10^−15^	6.83 × 10^−13^	9.39 × 10^−15^
mmu-miR-20b	1.07 × 10^−15^	6.83 × 10^−13^	9.39 × 10^−15^
mmu-miR-291a-3p	1.81 × 10^−12^	2.05 × 10^−13^	1.72 × 10^−14^
mmu-miR-294	1.81 × 10^−12^	2.05 × 10^−13^	1.72 × 10^−14^
mmu-miR-295	1.81 × 10^−12^	2.05 × 10^−13^	1.72 × 10^−14^
mmu-miR-302a	1.81 × 10^−12^	2.05 × 10^−13^	1.72 × 10^−14^
mmu-miR-302b	1.81 × 10^−12^	2.05 × 10^−13^	1.72 × 10^−14^
mmu-miR-302d	1.81 × 10^−12^	2.05 × 10^−13^	1.72 × 10^−14^
mmu-miR-128	9.36 × 10^−11^	5.70 × 10^−8^	4.81 × 10^−9^
mmu-miR-214	2.64 × 10^−10^	9.77 × 10^−8^	4.92 × 10^−6^
mmu-miR-761	2.64 × 10^−10^	9.77 × 10^−5^	4.92 × 10^−6^
mmu-miR-141	4.13 × 10^−10^	3.10 × 10^−8^	1.68 × 10^−8^
mmu-miR-200a	4.13 × 10^−10^	3.10 × 10^−8^	1.68 × 10^−8^
mmu-miR-203	1.33 × 10^−10^	3.22 × 10^−9^	4.17 × 10^−9^
mmu-miR-199a-5p	4.65 × 10^−7^	4.72 × 10^−5^	1.91 × 10^−5^
mmu-miR-200b	1.16 × 10^−6^	2.69 × 10^−6^	1.50 × 10^−6^
mmu-miR-200c	1.16 × 10^−6^	2.69 × 10^−6^	1.50 × 10^−6^
mmu-miR-429	1.16 × 10^−6^	2.69 × 10^−6^	1.50 × 10^−6^
mmu-miR-96	4.50 × 10^−6^	3.64 × 10^−6^	4.93 × 10^−7^
mmu-miR-130a	6.00 × 10^−6^	3.46 × 10^−5^	1.53 × 10^−5^
mmu-miR-301a	6.00 × 10^−6^	3.46 × 10^−5^	1.53 × 10^−5^
mmu-miR-130b	6.00 × 10^−6^	3.46 × 10^−5^	1.53 × 10^−5^

KS: Kolmogorov-Smirnov; *t*: Student *t*; Wilcoxon: Wilcoxon rank-sum test.

* *P*-values for the analysis of ES cell-trophoblast data are indicated.

**Table 3:** Highly ranked miRNAs which may be overrepresented in ES cells after differentiation to neural cells.

miRNA overrepresented after differentiation	*P*-values		

	KS	*t*	Wilcoxon
mmu-miR-184	0.809	0.006	0.985
mmu-miR-10b	0.876	0.039	0.996
mmu-miR-10a	0.876	0.039	0.996

KS: Kolmogorov-Smirnov; *t*: Student *t*; Wilcoxon: Wilcoxon rank-sum test.
